# Physical activity-related health competence differs by social and health-related factors: findings from a nationwide population-based study in Germany

**DOI:** 10.1186/s12889-026-29096-0

**Published:** 2026-08-19

**Authors:** Olga Maria Domanska, Susanne Jordan, Kristin Manz, Maike Buchmann, Klaus Pfeifer, Gorden Sudeck, Johannes Carl

**Affiliations:** 1 Department of Epidemiology and Health Monitoring, Robert Koch Institute, Berlin, Germany; 2 Department Sportwissenschaft und Sport, Friedrich-Alexander-Universität Erlangen-Nürnberg, Erlangen, Germany; 3 Department of Sports Science, Eberhard Karls Universität Tübingen, Tübingen, Germany; 4 Institute for Physical Activity and Nutrition, Deakin University, Geelong, Australia; 5 Department Health and Sport Sciences, Technical University of Munich, München, Deutschland

**Keywords:** PAHCO, Health literacy, Physical literacy, Adults, Health inequality, Health-related vulnerability, Social resources, Cross-sectional study, German Health Update Study (GEDA)

## Abstract

**Background:**

Insufficient physical activity remains a major public health challenge and is socially unequally distributed. Physical activity-related health competence (PAHCO) specifies the personal skills that enable health-enhancing physical activity. It integrates aspects of health literacy and comprises movement competence, control competence and self-regulation competence. This study examined the distribution of PAHCO in the adult population living in Germany and its associations with characteristics of social inequalities, health-related vulnerability, and limited social resources.

**Methods:**

We used data from a cross-sectional nationwide population-based survey of German-speaking adults conducted via standardised computer-assisted telephone interviews between January and May 2023 (*n* = 3,986). Bivariate and multiple logistic regression analyses were employed to examine the relationship between low PAHCO levels (overall score and its three sub-competencies) and characteristics of social inequalities, including sociodemographic factors (older age, female gender, lower education, and lower equivalent income), health-related vulnerability (poor self-rated general and mental health, chronic conditions), and social resources (low social support).

**Results:**

Overall, 38.2% of the population showed low/rather low PAHCO levels. Higher odds of low/rather low PAHCO were observed among adults aged 65 years and older compared to 18-29-year-olds (OR = 3.77, 95% CI: 2.28–6.22), among individuals with low versus high formal education (OR = 1.96, 95% CI: 1.37–2.82), and among those in the lowest versus highest income group (OR = 1.95, 95% CI: 1.23–3.10). The further relevant predictors of lower PAHCO were poor self-rated general, mental health and chronic conditions as well as low social support. These patterns were consistent across all three sub-competences, but age and gender differences were observed only for movement competence.

**Conclusion:**

The findings indicate a need to strengthen PAHCO in more than one third of the population and suggest that PAHCO could help reduce insufficient physical activity associated with social inequalities and health-related vulnerability. Strategies to promote movement competence, control competence, and self-regulation competence should particularly address the needs and living conditions of older adults, individuals with lower education levels, poorer health status, or lower social support. Physical activity promotion programmes should address all three sub-competences of PAHCO in a target-group-specific manner.

**Supplementary Information:**

The online version contains supplementary material available at 10.1186/s12889-026-29096-0.

## Background

Sufficient physical activity is a key factor in the prevention and treatment of non-communicable diseases [1]. However, the majority of the population in many countries, including Germany, does not achieve the necessary level of physical activity that is recommended by the World Health Organisation (WHO) [2, 3]. The WHO recommends that adults accumulate at least 150 min of moderate-intensity aerobic physical activity, or at least 75 min of vigorous-intensity aerobic physical activity (or an equivalent combination), together with muscle-strengthening activities involving all major muscle groups on at least two days per week [3]. The following population groups have been identified as being particularly prone to physical inactivity: women [4, 5], older adults [4, 6], adults with a low educational and income level [6, 7], individuals suffering from poor health [8–10] and groups experiencing lower levels of social support [11]. The promotion of physical activity is recognised as a key public health strategy, particularly for high-need population groups, in order to reduce health inequalities.

In strategic plans to promote physical activity and reduce non-communicable diseases, physical literacy and health literacy are identified as key components [1, 12]. The WHO sees the promotion of physical and health literacy as a goal of a comprehensive strategy to increase the physical activity of the population [1]. Most studies show a positive association between generic health literacy and physical activity behaviour, despite varied definitions, measurement instruments, and study populations [13–16]. In addition to the evolving research and practice in the domain of health literacy, the concept of physical literacy has gained increasing attention [17]. Physical literacy is regarded as a multidimensional concept that specifies the individual capabilities for physical activity throughout the life course, also serving as an educational goal for the work with children and adolescents [18, 19]. In addition to fundamental movement skills (functional physical level), individuals’ knowledge and understanding of the value of an active lifestyle (cognitive level) as well as the motivation and self-confidence (affective level) are regarded as pivotal components of physical literacy [20]. Another evolving concept that integrates ideas of physical literacy and health literacy is physical activity-related health competence (PAHCO), which has been primarily developed and applied in Germany and represents one of the concepts most closely related to physical literacy in the international literature. Nevertheless physical literacy and PAHCO are closely related but conceptually distinct frameworks [21]. While physical literacy broadly aims to foster lifelong engagement in diverse forms of physical activity, PAHCO explicitly focuses on developing the competencies required for health-enhancing physical activity. Thus, health is not merely one possible outcome or domain, but the overarching goal that shapes the construct. PAHCO is therefore conceptually located at the interface between physical literacy and health literacy by integrating motor-related, motivational, and cognitive capabilities required to engage in physical activity in ways that promote biopsychosocial health [22]. PAHCO has been more frequently applied among adults, as well as individual with chronic diseases [23] and comprises three sub-competences: movement competence, control competence (focusing on well-being and health) and self-regulation competence (focusing on motivation and volition), which are necessary in order to embed physical and sporting activity in everyday life in a way that is effective for health [23, 24]. PAHCO has been applied as a concept for promoting physical activity across different population groups at national and local levels, as well as in intervention contexts.

Information on the distribution of physical literacy respectively PAHCO are available for more and more countries [25]. Studies showed that physical literacy interventions have positive effects on several endpoints relevant to physical activity and health [26] thus also pointing to starting points for preventive public health strategies. In Germany, correlations between components of PAHCO and physical activity have been shown in various population groups using validated instruments [23, 27, 28]. First analyses with PAHCO and population-based data from Germany show a positive association between PAHCO and physical activity during leisure time, specifically people with high PAHCO levels compared with low/rather low levels of PAHCO have an almost four times higher likelihood to exert at least 2.5 h of physical activity per week [29]. The association was strongest for self-regulation competence, which appeared to be poorly distributed in the population living in Germany. Thus, it is important to understand which modifiable factors and resources promote higher levels of PAHCO and which population groups benefit from more support to enhance competences for health-enhancing physical activity.

So far, limited research has been conducted at the population level on PAHCO, physical literacy and physical activity among adults [16, 29]. Also, related predictors have not been sufficiently identified. Positive correlations have been described between physical activity and physical literacy as well as physical and motivational, but not cognitive components [30, 31]. However, a comprehensive understanding of the factors associated with the distribution and prevalence of PAHCO in Germany is essential for the development of effective strategies to strengthen the capabilities for life-long physical activity and identifying population groups with specific needs. Considering the socially unequal distribution of physical activity in Germany as mentioned above, it is imperative to gain a deeper understanding of how health inequities and health-related vulnerabilities are related to PAHCO in the population, considering age, education level, and health status.

The aim of our study is to examine whether PAHCO and its three sub-competences (movement competence, control competence and self-regulation competence) are associated with sociodemographic factors (age, gender, education, income), health-related characteristics (self-reported health status, mental health status, and chronic disease), and social resources (general social support). The findings of this study aim to inform the development of public health strategies to promote physical activity at a population level.

## Methods

### Study design and setting of the study

We analysed data of the survey „German Health Update 2023“ (GEDA 2023) [in German: “Gesundheit in Deutschland aktuell (GEDA)]”, which is regularly conducted by the Robert Koch Institute as a component of the nationwide population-based health monitoring programme in Germany [32, 33]. GEDA 2023 is a telephone-based survey using a random sample of the German speaking population aged 18 and older living in private households in Germany that can be reached via landline or mobile phone [32]. The sampling of landline and mobile numbers (dual-frame method) was based on the telephone sampling system of the German Business Association for Market and Social Research (ADM), which includes all possible telephone numbers in Germany [34]. The cross-sectional survey was conducted between January and May 2023 (*n* = 3,986) via telephone interview using a programmed, fully structured questionnaire (Computer Assisted Telephone Interview, CATI). All collected data were based on self-reports. The response rate, calculated in according to the standards of the American Association for Public Opinion Research (AAPOR) [35], was approximately 19%. The reporting of this study followed the Strengthening the Reporting of Observational studies in Epidemiology (STROBE) guide [36].

### Study variables

#### Outcome: Physical activity-related health competence

We measured physical activity-related health competence using the recently validated short version of the Physical Activity-related Health Competence questionnaire – PAHCO_12 (Supplementary Table S1). The instrument fulfils the reliability criteria for internal consistency (McDonald’s ω = 0.78–0.84), and acceptable factorial validity (CFI = 0.924, RMSEA = 0.073, SRMR = 0.044). The latent PAHCO dimensions showed significant associations with physical activity (β = 0.20–0.27) and self-rated health (β = 0.50–0.65) supporting criterion validity [37]. The PAHCO_12 instrument contains 12 items and was derived from the long version of the questionnaire (PAHCO) [37], which was developed for and validated across different areas of health promotion, prevention, and rehabilitation [23, 28]. In order to describe the distribution of the PAHCO and its sub-competences in the general population, the overall score and the sub-scores of the corresponding sub-competences were divided into four levels for the overall score and two levels for each sub-competence (Table 1).

**Table 1:** Definition and categorization of the PAHCO overall score and the sub-scores

Operationalised dimension	Definition according to the PAHCO model ^a^	Formula ^c^	[Range of the scores], threshold values for categorisation
Overall PAHCO score	Physical activity health-related competence comprises three sub-competences: movement competence, control competence and self-regulation competence.	Item_1INV^b^ + Item_2INV^b^ + Item_3 + Item_4 + Item_5 + Item_6 + Item_7 + Item_8 + Item_9 + Item_10 + Item_11 + Item_12	[0–48]; 21, 33, 42 0 ≤ low < 21 21 ≤ rather low < 33 33 ≤ rather high < 42 42 ≤ high ≤ 48
Movement competence	Motor skills and abilities, including body and movement perception and sensorimotor control necessary for coping with the motor requirements of health-enhancing physical activity. People with a high level of movement competence are able to perform a variety of health-enhancing physical and sporting activities.	Item_1INV^b^ + Item_2INV^b^ + Item_3 + 0.5 x Item_4 + 0.5 x Item_9	[0–16], 11 0 ≤ (rather) low < 11 11 ≤ (rather) high ≤ 16
Control competence	The ability to manage physical exertion based on knowledge of physical and sporting activities (e.g. knowledge of training methods, exertion dosage, effects on health and well-being) and with the help of refined bodily and movement awareness (e.g. movement execution, heart rate, perceived exertion, affective state), thereby avoiding over- or under-exertion as well as discomfort or inappropriate physical exertion.	0.5 x Item_4 + Item_5 + Item_6 + Item_7 + Item_8	[0–18], 12.375 0 ≤ (rather)low < 12.375 12.375 ≤ (rather) high ≤ 18
Self-regulation competence	The ability to set personal goals for health-enhancing physical activity based on intrinsic motivation and then implement these goals regularly in everyday life, even when obstacles (e.g. bad weather) and competing intentions (e.g. watching favourite TV show) arise. This sub-competence of the PAHCO builds on motivational and volitional determinants of physical activity behaviour (e.g. attitudes, motivational structure, self-efficacy).	0.5 x Item_9 + Item_10 + Item_11 + Item_12	[0–14]; 9.625 0 ≤ (rather) low < 9.625 9.625 ≤ (rather) high ≤ 14

Legend: ^a^ Sudeck et al. 2016 [23] and Geidl et al. (2026) [38]; ^b^ Items 1 and 2 must be inverted; ^c^ before forming the scale, all items must be transformed from 1 to 5 into 0–4 (which means a subtraction of 1 for each item)

The corresponding threshold values were based on the combination of content-related criteria and on the comparison of item difficulties in several populations (from a data pooling study: [39]). Content-related criteria refer to the response options that were explicitly formulated along the response spectrum for each competence. For example, the ‘competence’ of being able to perform strenuous endurance activities (slow jogging) or carrying a 10 kg shopping bag over several floors could be rated “without problems” or only “with slight difficulties” (in line with the response options), providing orientation for the determination of the thresholds. The items showed a tendency toward higher response categories resulting in relatively high ratings of physical activity-related abilities and skills and a ceiling effect. Due to the relatively low item difficulty (skewed item distribution), more stringent thresholds were set. For each subscale, the threshold value per item was set at a minimum value of 2.75 (with a potential range between 0 and 4). The calculation formula of the overall score and the sub-scores with corresponding thresholds can be found in Table 1 also and in the initial validation study [37].

#### Sociodemographic factors

The data on *age* was categorized into four age groups: 18–29, 30–44, 45–64 years old, and 65 and older.

In the analyses, *gender* was recognised as a socially defined concept gathered through self-report/identity [40] with the respondents’ categorisation into ‘male’, ‘female’, and ‘diverse’. Due to the small number of cases (*n* = 17), persons with diverse gender identities were excluded from the analysis due to insufficient statistical power. In the case of a further 17 people, their sex registered at birth (proxy for biological sex) did not correspond with their gender identity. Due to the small number of transgender respondents in the sample, we did not expect effects on the outcome variables, and these 17 individuals remained in the sample. 

Education and vocational qualifications were recorded according to the CASMIN (Comparative Analysis of Social Mobility in Industrial Nations) classification [41, 42] with the following question: “*What is your highest level of vocational training or university/university of applied sciences qualification*?”. The levels of education were assigned to three categories: ‘low’, ‘medium’, and ‘high’ education group. Net equivalent *income* was recorded with the question: “*What is your household’s total monthly net income? This refers to the sum resulting from wages*,* salary*,* freelancer income*,* pensions or retirement benefits. Please also include income from public benefits*,* income from letting*,* leasing*,* housing benefits*,* child benefits and other income. Then deduct taxes*,* operating expenses and social security contributions*.“ Missing income information was imputed using regression-based methods with information on age, gender, composition of the household, education, occupational position and regional information on unemployment and income tax. The equivalent income was divided into quintiles.

#### Health-related factors

*Self-rated general health* was recorded using questions from the Minimum European Health Module [43], which is a central component of all national health surveys in the EU: *“How is your health in general*?”. Participants were asked to choose one of five response options: ‘very good’, ‘good’, ‘fair’, ‘bad’ or ‘very bad’. As the population-based health monitoring and surveillance of noncommunicable diseases defines the answers ‘very good’ or ‘good’ as a positively perceived general health, we dichotomised answers in two levels: ‘very good/good’ versus ‘very bad/bad/fair’.

*Self-rated mental health* was reported via the question: “*How would you describe your mental health in general?”.* Participants could choose one of the five response options: ‘excellent’, ‘very good’, ‘good’, ‘fair’ and ‘poor‘. We summarized answers to three levels: ‘very good/excellent’, ‘good’ and ‘poor/fair’. The categorization of the self-rated general and mental health variables was informed by their observed response distributions. Because the extreme response categories were only rarely selected and adjacent categories showed similar associations with the PAHCO outcomes, response categories were combined where appropriate to ensure meaningful group sizes and stable regression estimates.

The indicator for *chronic conditions* was collected via the question: “*Do you have any chronic disease or a long-term health problem? This means diseases or health problems that have lasted or are expected to last for at least 6 months*”. The response options were ‘yes’, ‘no’, or ‘don’t know’, the latter coded as missing values.

#### Social resources

Social support is defined as the perceived availability of people whom the individual trusts and who make one feel cared for, loved, esteemed and valued as a person [44]. Social support is determined by factors at the individual as well as the social level. The Oslo Social Support Scale (OSSS-3) was used to measure the subjective perceived availability of *social support* [43, 45]. OSSS-3 is a composite scale measuring perception of both support and social network. We asked following three questions: “*How many people are so close to you that you can count on them if you have serious personal problems*?” (response option: ‘none’ (1), ‘1 to 2’ (2), ‘3 to 5’ (3), ‘6 or more’ (4)), “*How much concern do people show in what you are doing*?” (response option: ‘a lot of concern and interest’ (5), ‘some concern and interest’ (4), ‘uncertain’ (3), ‘little concern and interest’ (2), ‘no concern and interest’ (1)) and “*How easy is it to get practical help from neighbours in case of need*?” (response option: ‘very easy’ (5), ‘easy’ (4), ‘possible’ (3), ‘difficult’ (2), ‘very difficult’ (1)). By adding the individual scores from the three questions, an index was formed resulting in a potential range between 3 and 14 points. If one of the three variables contained a missing value, the index was missing also. The range of 3 to 8 points was considered poor support, 9 to 11 points moderate support, and 12 to 14 points strong support [46].

### Statistical analyses

The analyses were carried out using a weighting factor to correct for deviations of the sample from the population structure (design and adjustment weighting). More specifically, design weighting was first carried out for the different selection probabilities (mobile and landline numbers). The adjustment weighting variable refers to the population status as at 31 December 2020 supplied by the Federal Statistical Office and the education weighting is based on the 2018 micro-census (sample census) in Germany [32]. It corrects for deviations of the sample from the population according to sex, age, education and place of residence.

Bivariate and multivariate analyses were used to analyse the association between indicators of physical activity-related health competence (i.e., overall score and the three sub-competences) and social inequalities, health-related vulnerability, and limited social resources. These characteristics were: (1) gender, (2) age group, (3) education, (4) income, (5) self-rated general health, (6) chronic conditions, (7) self-rated mental health, and (8) social support (independent variables). For this purpose, the PAHCO overall score and the three sub-scores were dichotomized at the outcome level. In initial bivariate analyses, we examined how the PAHCO dimensions were most associated with sociodemographic characteristics. We conducted complete case analyses for all bivariate analyses and main models. Before performing the multivariate analysis, Spearman’s rho (*ρ*) was used to measure the strength and direction of the relationship between all the independent variables. The odds ratio (OR) for ‘low/ rather low’ levels of the PAHCO indicator, including their 95% confidence intervals (95% CI), were calculated as part of multiple binary logistic regression models. All independent variables were entered in the model simultaneously. Variance inflation factors (VIF) were calculated to examine potential multicollinearity, indicating how much the variance of a regression coefficient is inflated by correlations with other independent variables. Values of VIF exceeding 10 are often regarded as indicating multicollinearity. However, in weaker models — which is often the case in logistic regression and in our model — values above 2.5 may be cause for concern [47]. An F-adjusted mean residual goodness-of-fit test was applied to evaluate the overall model fit [48]. Additionally, we determined adjusted McFadden’s R^2^ to quantify the overall explained variance of the entire logistic regression. A value between 0.2 and 0.4 is often considered acceptable in fields like social sciences and economics [49]. For all analyses, the level of significance was set to p-values < 0.05.

Additionally, we explored two interaction terms (gender x age as well educational achievement x age group) in the adjusted models. These interactions were selected based on previous evidence suggesting that gender differences in physical activity may vary across age groups because of cohort-related differences in opportunities for lifelong physical activity [4, 5], and that educational inequalities in physical activity may accumulate over the life course [6]. Each interaction was added separately to the fully adjusted weighted logistic regression models. Joint significance of the interaction terms was assessed using design-based Wald tests. Finally, we conducted a robustness analysis using metric outcomes for all models. We performed data preparation and statistical analyses using the statistic software STATA^®^ version 19 (StataCorp LLC, College Station, TX, USA).

## Results

### Sample

The total sample comprising *n* = 3,986 individuals included 51.5% women and 48.5% men. The participants were, on average, 51.7 [95% CI 50.7–52.8] years old. A total of 644 individuals (26.7%) were in the low education group, 1,793 individuals (54.6%) in the medium education group, and 1,542 individuals (18.7%) in the high education group. Furthermore, 1,322 participants (35.3%) reported very bad, bad, or fair general health, whereas 2,659 participants (64.7%) reported good, or very good general health. 2,091 individuals (52.0%) indicated to have at least one chronic condition, while 1,881 individuals (48.0%) indicated to have no chronic condition. More detailed information about the sample (e.g., income, health-related factors, social resource, and PAHCO) can be found in Table 2.

38.2% in the German population have a ‘low’ (13.4%) or ‘rather low’ (24.7%) level of PAHCO. In terms of sub-competences, we observed the following proportions of people with a ‘low/rather low’ level: 32.7% for movement competence, 40.0% for control competence, and 49.3% for self-regulation competence.

**Table 2:** Sample description and proportions of the physical activity-related health competencies in German population (GEDA 2023, *n* = 3,986)

		%	[95% CI]	*n* (%)
		weighted	weighted	unweighted
**PHYSICAL ACTIVITY-RELATED HEALTH COMPETENCE**				
Overall PAHCO score (categorized)	Low	13.4	[11.7–15.3]	444
	Rather low	24.7	[22.6–27.0]	973
	Rather high	39.7	[37.2–42.2]	1,543
	High	22.1	[20.2–24.2]	958
	Missings			68 (1.71)
Overall PAHCO score (dichotomized)	Low/rather low	38.2	[35.7–40.7]	1,417
	Rather high/high	61.8	[59.3–64.3]	2,501
	Missings			68 (1.71)
Movement competence	Low/rather low	32.7	[30.4–35.2]	1,267
	Rather high/high	67.3	[64.8–69.6]	2,689
	Missings			30 (0.75)
Control competence	Low/rather low	40.0	[37.6–42.6]	1,404
	Rather high/high	60.0	[57.4–62.4]	2,510
	Missings			72 (1.81)
Self-regulation competence	Low/rather low	49.3	[46.8–51.8]	1,850
	Rather high/high	50.7	[48.2–53.2]	2,085
	Missings			51 (1.28)
**SOCIODEMOGRAPHIC CHARACTERISTICS**				
Gender	Female	51.5	[48.9–54.0]	2,111
	Male	48.5	[46.0-51.1]	1,858
	Missings			17 (0.43)
Age groups	18–29 years	16.0	[13.9–18.3]	312
	30–44 years	22.8	[20.7–25.2]	598
	45–64 years	34.6	[32.3–36.9]	1,500
	≥ 65 years	26.6	[24.7–28.7]	1,576
	Missings			0 (0)
Education	Low	26.7	[24.4–29.2]	644
	Medium	54.6	[52.1–57.1]	1,793
	High	18.7	[17.2–20.2]	1,542
	Missings			7 (0.18)
Equivalent income	1st Quintile (lowest)	21.0	[18.8–23.5]	482
	2nd Quintile	19.7	[17.7–21.8]	670
	3rd Quintile	18.3	[16.5–20.2]	792
	4th Quintile	19.3	[17.5–21.2]	873
	5th Quintile (highest)	21.7	[19.8–23.6]	1,169
	Missings			0 (0)
**HEALTH-RELATED FACTORS**				
Self-rated general health	Very bad/bad/fair	35.3	[33.0-37.8]	1,322
	Good/very good	64.7	[62.2–67.0]	2,659
	Missings			5 (0.13)
Chronic condition	Yes	52.0	[49.5–54.5]	2,091
	No	48.0	[45.5–50.5]	1,881
	Missings			14 (0.35)
Self-rated mental health	Poor/fair	17.5	[15.5–19.6]	517
	Good	47.0	[44.6–49.6]	1,862
	Very good/excellent	35.5	[33.2–37.8]	1,598
	Missings			9 (0.23)
**SOCIAL RESOURCE**				
Social support	Poor	17.2	[15.2–19.4]	461
	Moderate	47.6	[45.1–50.1]	1,816
	Strong	35.2	[32.9–37.5]	1,598
	Missings			111 (2.78)

Legend: *CI* Confidence interval

### Association analyses

Based on bivariate analyses with the sociodemographic characteristics (Table 3), age and educational attainment were found to be most strongly associated with PAHCO. The proportion of rather low competence increased with age and decreased with higher education across all competence domains, most markedly for movement competence (9.8% among adults aged 18–29 years vs. 54.7% among those aged 65 years and over; 15.1% in the high education group vs. 54.7% in the low education group). Similar but less pronounced gradients were observed for the overall PAHCO score and control competence. Self-regulation competence showed comparatively small sociodemographic differences, and no significant gender differences were observed.

**Table 3:** Proportion of the German population with (rather) low PAHCO levels by sociodemographic characteristics (GEDA 2023; *n* = 3,845)

	Sociodemographic characteristics	PAHCO overall	Movement competence	Control competence	Self-regulation competence
		% (rather) low [95% CI]	% (rather) low [95% CI]	% (rather) low [95% CI]	% (rather) low [95% CI]
**Gender**	Gender				
	Female	40.9 [37.5–44.4]	36.7 [33.4–40.2]	42.1 [38.6–45.6]	50.8 [47.3–54.3]
	Male	34.9 [31.4–38.5]	27.1 [23.8–30.5]	36.9 [33.3–40.6]	47.4 [43.7–51.1]
	p-value	0.018	< 0.001	0.043	0.195
**Age groups**	18–29 years	19.8 [14.3–26.8]	9.8 [5.7–16.3]	31.3 [24.6–39.0]	35.7 [28.8–43.2]
	30–44 years	27.4 [22.2–33.3]	18.2 [13.5–24.0]	29.4 [24.2–35.3]	46.6 [40.7–52.5]
	45–64 years	39.5 [35.7–43.5]	35.2 [31.4–39.1]	38.5 [34.7–42.5]	52.0 [48.2–55.8]
	65 + years	57.1 [52.9–61.2]	54.7 [50.4–58.9]	55.5 [51.3–59.6]	56.2 [51.9–60.3]
	p-value	< 0.001	< 0.001	< 0.001	< 0.001
**Education**	Low	58.5 [52.7–64.1]	54.7 [49.0–60.4]	57.5 [51.8–63.1]	60.8 [55.1–66.3]
	Medium	33.9 [30.7–37.3]	27.2 [24.3–30.4]	36.6 [33.3–40.1]	46.3 [42.9–49.9]
	High	21.7 [18.9–24.8]	15.1 [13.1–17.4]	23.7 [20.6–27.0]	41.3 [37.5–45.2]
	p-value	< 0.001	< 0.001	< 0.001	< 0.001
**Equivalent income**	1st Quintile (lowest)	53.1 [46.3–59.7]	46.4 [39.8–53.2]	54.0 [47.3–60.6]	55.6 [48.8–62.2]
	2nd Quintile	41.9 [36.1–47.9]	34.7 [29.4–40.5]	42.3 [36.5–48.4]	49.6 [43.6–55.7]
	3rd Quintile	42.3 [36.9–47.8]	33.0 [28.1–38.2]	42.4 [36.9–48.0]	58.0 [52.6–63.3]
	4th Quintile	31.2 [26.6–36.2]	26.8 [22.4–31.8]	35.5 [30.6–40.7]	41.5 [36.5–46.7]
	5th Quintile (highest)	22.8 [19.3–26.8]	20.1 [16.5–24.1]	24.9 [21.0–29.3]	41.9 [37.4–46.7]
	p-value	< 0.001	< 0.001	< 0.001	< 0.001

Legend: *CI* Confidence interval

Further, initial bivariate association analyses revealed that the eight independent variables are weakly associated with each other (|0.02 ≤ *ρ* ≤ 0.22|), except of the moderate associations of education with income (*ρ* = 0.34) and of health status with chronic disease (*ρ* = 0.43) and mental health (*ρ* = 0.41) (for details, see Supplementary Table S2). Accordingly, we did not detect any severe case of multicollinearity (VIF ≤ 1.5; Mean VIF < 1.2) and we, therefore, did not exclude any sociodemographic, health-related, or social factor for the multivariate associations with PAHCO.

Table 4 shows results from the multivariable regression analyses. Individuals aged between 45 and 64 years (OR = 2.06) as well as 65 years and older (OR = 3.77) had a significantly higher risk of being classified as having rather low or low overall PAHCO score than individuals between 18 and 29 years. Similarly, participants from the lowest (OR = 1.95) and the middle-income quintile (OR = 1.83) had a higher risk than participants from the highest-income quintile. Moreover, participants in the low education group (OR = 1.96) had a higher risk for rather low or low PAHCO than participants in the high education group. Both self-rated general health and chronic conditions discriminated between people having low/rather low PAHCO and rather high/high PAHCO, registering higher risks of very bad/bad/fair (OR = 3.64) self-rated general health and the presence of a chronic condition (OR = 1.70) for unfavourable PAHCO levels. A poor/fair self-rated mental health revealed a four times (OR = 4.20) and good mental health (OR = 2.04) a two times higher chance for showing low/rather low PAHCO as compared to a very good/excellent mental health. Finally, poor social support (OR = 2.22) was associated with a significantly higher risk of being classified as having low/rather low PAHCO compared to strong social support. Gender was not associated with PAHCO in our sample. The adjusted McFadden R^2^ = 0.28 with eight variables shares a moderate amount of variance in PAHCO. According to results of the F-adjusted mean residual test that assess goodness of fit of a design-based logistic regression models, all models demonstrated a good fit.

**Table 4:** Associations between PAHCO and the factors considered. Results of the multivariable logistic regression analyses (GEDA 2023, *n* = 3,734)

		PAHCO (low/rather low vs. rather high/high (ref.))			
		Overall score dichotomized	Movement competence	Control competence	Self-regulation competence
		OR [95% CI]	OR [95% CI]	OR [95% CI]	OR [95% CI]
**Gender**	Female	1.20 [0.92–1.56]	1.65 [1.23–2.23]***	1.15 [0.90–1.48]	1.08 [0.87–1.35]
	Male	ref.	ref.	ref.	ref.
**Age group**	18–29 years	ref.	ref.	ref.	ref.
	30–44 years	1.29 [0.74–2.25]	1.75 [0.77–3.97]	0.71 [0.43–1.17]	1.34 [0.86–2.08]
	45–64 years	2.06 [1.24–3.43]**	4.40 [2.18–8.88]***	0.96 [0.61–1.52]	1.44 [0.96–2.16]
	65 + years	3.77 [2.28–6.22]***	9.49 [4.70-19.16]***	1.55 [0.97–2.48]	1.33 [0.88–2.03]
**Education**	Low	1.96 [1.37–2.82]***	2.99 [2.06–4.35]***	1.92 [1.36–2.70]***	1.27 [0.91–1.76]
	Medium	1.24 [0.95–1.63]	1.52 [1.14–2.03]**	1.31 [1.00-1.71]	0.97 [0.76–1.24]
	High	ref.	ref.	ref.	ref.
**Equivalent income**	1st Quintile (lowest)	1.95 [1.23–3.10]**	1.84 [1.11–3.04]*	1.67 [1.10–2.54]*	1.00 [0.66–1.50]
	2nd Quintile	1.31 [0.86-2.00]	0.91 [0.58–1.44]	1.26 [0.85–1.86]	0.93 [0.65–1.32]
	3th Quintile	1.83 [1.24–2.71]**	1.21 [0.80–1.84]	1.64 [1.13–2.40]*	1.61 [1.16–2.24]**
	4th Quintile	1.09 [0.74–1.59]	1.02 [0.68–1.52]	1.31 [0.90–1.89]	0.81 [0.59–1.11]
	5th Quintile (highest)	ref.	ref.	ref.	ref.
**Self-rated general health**	Very bad/bad/fair	3.64 [2.71–4.91]***	3.80 [2.66–5.43]***	2.88 [2.14–3.87]***	2.58 [1.94–3.43]***
	Good/very good	ref.	ref.	ref.	ref.
**Chronic condition**	Yes	1.70 [1.29–2.25]***	2.26 [1.61–3.17]***	1.36 [1.03–1.79]*	1.31 [1.03–1.66]*
	No	ref.	ref.	ref.	ref.
**Self-rated mental health**	Poor/fair	4.29 [2.72–6.77]***	4.20 [2.48–7.11]***	3.25 [2.17–4.86]***	2.13 [1.42–3.22]***
	Good	1.94 [1.47–2.58]***	2.04 [1.50–2.77]***	2.17 [1.66–2.84]***	1.42 [1.11–1.81]**
	Very good/excellent	ref.	ref.	ref.	ref.
**Social support**	Poor	2.22 [1.44–3.44]***	1.63 [1.02–2.60]*	2.03 [1.37-3.00]***	1.76 [1.23–2.52]**
	Moderate	0.93 [0.71–1.21]	0.96 [0.71–1.29]	1.02 [0.78–1.32]	1.18 [0.93–1.48]
	Strong	ref.	ref.	ref.	ref.
**Model fit**	Adjusted McFadden R^2^	0.28	0.34	0.19	0.11

Legend: *OR *Odds ratio, *CI *Confidence interval, *Ref*. reference group, *p < 0.05; **p < 0.01; ***p < 0.001

Upon inspecting the three sub-competences of PAHCO, we identified similar associative patterns. More specifically, *movement competence* yielded the same results (regarding statistical significance) for age, self-rated general health, chronic conditions, self-rated mental health, and social support (adj. McFadden *R*² = 0.34). In contrast to the overall score, a gender difference was observed, with women demonstrating a higher risk for having low/rather low movement competence than men (OR = 1.65). Additionally, regarding income, only the lowest quintile (OR = 1.84) –and not also the middle quintile – had a higher risk for low/rather low movement competence as compared to the highest income quintile. We also recorded a stronger education gradient with higher risks of low/rather low movement competence evident in both the low (OR = 2.99) and the medium (OR = 1.52) education groups relative to the high education group.

When defining *control competence* as the outcome (adj. McFadden *R*² = 0.19), the following groups had a higher probability for showing low values relative to their reference groups: low education (OR = 1.92) relative to high education, the lowest (OR = 1.67) and middle (OR = 1.64) income quintiles relative to the highest quintiles, very bad/bad/fair self-rated general health (OR = 2.88) relative to good/very good general health, the presence of a chronic condition (OR = 1.36) relative to its absence, poor/fair self-rated mental health (OR = 3.25) and only good mental health (OR = 2.17) relative to very good/excellent mental health, and poor social support (OR = 2.03) relative to high support. Gender and age were no significant correlates of control competence.

A higher risk for low/rather low *self-regulation competence* was observed among individuals in the middle-income group (OR = 1.61) as compared to those in the highest income group. Again, the report of a very bad/bad/fair self-rated general health (OR = 2.58), a chronic condition (OR = 1.31), a poor/fair (OR = 2.13) or good self-reported mental health (OR = 1.42), and poor social support (OR = 1.76) were all significant correlates for low/rather low self-regulation competence relative to their reference groups. Gender, age, and education were non-significant variables in the multivariate model of self-regulation competence (adj. McFadden *R*² = 0.11).

No statistically significant interaction was observed between gender and age group for the overall PAHCO score (global Wald test: F(3,3731) = 1.08, *p* = 0.356) or for any of the three sub-competencies (all *p* ≥ 0.142). Likewise, there was no evidence of interactions between age group and educational attainment (all *p* ≥ 0.149). These findings indicate that the associations of age, gender, and educational attainment with PAHCO were consistent across the examined subgroups (Supplementary Table S3).

The robustness analyses using continues PAHCO scores largely confirmed the findings of the main logistic regression analyses. The direction and relative strength of the associations remained highly consistent across all outcomes. Although four previously non-significant or borderline associations reached statistical significance when the continuous outcomes were used, the overall pattern of results remained unchanged, indicating that the main findings are robust and not driven by the dichotomization of the PAHCO outcomes (Supplementary Table S4).

## Discussion

The study examined the associations between socio-demographic characteristics (as indicators of social inequalities), health-related vulnerability, and low social resources with indicators of PAHCO, which comprise the ability to engage in physical activity in a health-enhancing way. In our nationwide population-based study, we found that low PAHCO levels concern almost 4 in 10 people in Germany, and those levels are more prevalent in some population groups, including older persons, persons with low education, and from the lowest-income groups. However, the strongest correlates of lower PAHCO levels were health-related factors, including rather poor self-rated general and mental health and having a chronic condition. Likewise, low social support was associated with lower levels of PAHCO.

Similar association patterns to those revealed by the overall PAHCO were observed for movement competence. However, women had a statistically significantly higher risk of low movement competence than men. This risk was also higher among people with lower levels of education. Neither gender, age or income were identified as sociodemographic predictors for control and self-regulation competence. In contrast, poor health-related indicators and low social support were significantly associated with low levels of all three PAHCO sub-competences.

The PAHCO concept has been examined primarily in German-speaking countries (Germany, Austria and Switzerland), with most studies conducted in Germany [50, 51]. Comparing our findings for the population in Germany with those of other studies is limited by two methodical differences. First, we used a short instrument in a population-based sample [37] as opposed to other PAHCO studies in German-speaking countries which mostly used a long version of the instrument. Second, the previous study samples were smaller and/or more homogeneous, originating from the prevention or rehabilitation sectors [39]. Furthermore, when comparing with other international studies on physical literacy, the concepts of the PAHCO and physical literacy as mentioned before, slightly differ regarding the definitions, components and operationalisation [51].

Our study found that older age consistently was a risk factor for lower PAHCO levels. This finding strongly aligns with a data pooling study on PAHCO (with samples from Germany, Switzerland and Austria) for movement competence [39], supporting the strong evidence from physical activity research about a decline in physical activity during the second half of the lifespan [4, 6]. These patterns may be explained by age-related physiological and functional changes [52, 53]. In contrast to our findings, in which no age difference regarding control and self-regulation could be observed, the findings from the data pooling study indicated that these sub-components were modelled to remain stable or even slightly improve with age [39]. The differences might be explained by the fact that the authors included many studies from the rehabilitation sector, with the non-representative sample missing participants from older age or accumulating many participants who might have learned over time to positively use physical activity in the context of their disease [39]. In this regard, our study adds evidence to individual determinants of health-enhancing physical activity on a population-level, which is limited for older adults. The higher risk of a low/rather low movement competence in older adults supports calls for an increasing shift to adapting movement, maintaining function, and knowledge about age-related changes rather than maximizing performance [54, 55]. More specifically, the goal should be to maintain individuals’ confidence and capability by focusing not only on their fitness progress, but also on identifying the most appropriate social context and physical activity preferences for older age (e.g., to reduce social isolation [56, 57]).

Additionally, we found that persons with low education reported more frequently lower level of movement competence and control competence. The finding related to control competence may be explained by limited access to educational opportunities to learn about healthy exercise methods or training principles, e.g. in sports clubs. In this regard, PAHCO shows strong conceptual overlaps with generic health literacy, which has been largely shown to follow educational gradients [58, 59]. However, the present study also revealed an educational gradient for movement competence, which is the theoretical component that acknowledges the fact that physical activity strongly involves the body and its motor system. Regarding this sub-component of PAHCO, empirical parallels cannot be drawn from the existing health literacy literature, as the physical aspect is not a prominent part of current conceptualisations and operationalisations. Here an educational gradient was found despite controlling for aspects, such as health status, disease, and age. One explanation for this could be that people with lower levels of formal education are less physically active in leisure-time and engage less in health-enhancing physical activity [60]. They more frequently perform occupational work that is often repetitive, externally paced, below the necessary thresholds for enhancing functional or cardiorespiratory capacity, and therefore not necessarily health-promoting [61]. Those groups also have less access to sports programs, sport infrastructure and green spaces, both representing financial and environmental barriers. These structural barriers may limit opportunities to develop movement competence.

One very consistent finding across all PAHCO components was that people with poor self-reported general or mental health and chronic conditions more often reported low levels of the PAHCO. Links between PAHCO and physical health as well as health-related quality of life, subjective vitality, and perceived fitness were also found, in previous studies applying the PAHCO measure on prevention samples such as office workers (intervention study) or participants of sport courses at universities [27, 62, 63]. Nevertheless, the cross-sectional design of the present study does not allow for any conclusions to be drawn about a direction of the found associations between lower level of PAHCO and poor self-rated health and chronic conditions. From the theoretical perspective we assume a lower level of the PAHCO results in less physical activity and contribute to poorer health-related outcomes and higher risk of chronic diseases [64]. Conversely, strengthening PAHCO may increase physical activity levels in the population, whose preventive effect on chronic diseases are well-established in public health research [65]. In addition, associations between mental health and PAHCO observed in our study were found in an intervention study [66]. In summary, this representative sample highlights the need to support individuals with mental health issues [67, 68], with PAHCO advocating for a balanced consideration of physical, affective, and cognitive experiences.

The present study suggested that social support might facilitate individuals’ specific competence for health-enhancing physical activity. Social support – provided by family, peers, teachers, and community members – is widely acknowledged as a critical interpersonal and social environmental influence of leisure-time physical activity behaviours [11]. An observational, longitudinal, multilevel study controlled for age demonstrated an association between social support and physical activity, accounting for sociodemographic and health variables [69]. In addition, key research already emphasised the potential critical role of social support for generic health literacy, for example, through opportunities to engage with others, ask questions, and apply health information with peers [70]. Latest evidence fuelled this assumption, demonstrating empirical associations between social support and health literacy [71]. Paralleled by findings showing a link between physical literacy and social support in studies with adolescents and patients with chronic diseases [72, 73]. To our knowledge, this is the first population-based study demonstrating associations between social support and PAHCO, including all three sub-competences. PAHCO might therefore represent an important mechanism linking social support and physical activity behaviour.

### Methodical strengths and limitations

This study is based on the first population-wide data on PAHCO for Germany. The large and representative population-based sample (*n* ~ 4000) enhances the generalizability of our findings regarding social and health-related correlates of PAHCO in the adult population living in Germany. We used the short version of PAHCO questionnaire [37]. This instrument is grounded in the complex model that combines the physical, cognitive and motivational aspects of health-enhancing physical activity. It has proven psychometric properties and only takes a few minutes to complete.

However, several limitations must be acknowledged. Firstly, data collection was conducted via telephone interviews, which may have resulted in socially desirable response behaviour and reporting bias. Participants might have overreported their PAHCO levels or underreported the sensitive topics such as general and mental health. Secondly, selection bias cannot be ruled out. People with lower levels of education, who are older and who have severe illnesses tend to participate less frequently in health surveys. Consequently, these groups may therefore be underrepresented in our sample. To mitigate this bias, we applied established sampling and weighting procedures to ensure a more balanced representation of the population [32]. Despite these efforts, some level of underrepresentation may persist, limiting the extent to which the findings can be generalized.

Thirdly, the study was restricted to German-speaking individuals and, therefore, does not reflect the linguistic and cultural diversity within the entire population. Finally, causality cannot be inferred due to the cross-sectional study design. Despite these limitations, the study provides population-based evidence on PAHCO and benefits from its large, population-based sample, methodological rigour, and the use of validated measures.

### Implications for research and public health

The present findings show that the inequalities in physical activity are also observable in the competences required to engage in life-long health-enhancing physical activity. This emphasises importance of the PAHCO concept in understanding and promoting health-enhancing physical activity within the population. Similar to physical literacy, PAHCO specifies individual prerequisites for a physically active lifestyle that go beyond the access, understanding, and application of health information typically addressed in health literacy research [74, 75]. The high proportion of individuals with low or rather low PAHCO levels in the population living in Germany indicates a need for action. Strategies to promote movement competence, control competence, and self-regulation competence should address the contexts and living conditions of the whole population and especially of older population groups, those with lower levels of education, poorer physical or mental health, and less social support. Considering the low level of PAHCO in the general adult population in Germany, it is crucial to implement structural prevention measures regarding PAHCO at all levels, from children to the elderly. Alongside structural and infrastructural measures, this should include educational strategies and societal conditions that make it easier to acquire PAHCO. Given the observed differences across the three PAHCO sub-competences, interventions should adopt a more tailored approach, or at least a group-specific one, and address the different competence dimensions integratively. This may be relevant across different sectors, including prevention, rehabilitation, education, and community sport settings.

From a research perspective, longitudinal studies are needed to improve our understanding of the causal pathways between PAHCO and physical activity, and of how single sub-competences develop across the life course. Additionally, intervention studies should examine whether strengthening PAHCO leads to sustained increases in physical activity and improvements in health outcomes. Finally, repeated population-based survey of PAHCO within national health surveillance systems could help monitor developments over time and contribute to evaluation of the effectiveness of national strategies and interventions aimed at reducing physical inactivity [25].

## Conclusion

PAHCO reflects how well people can draw on their physical, cognitive, and affective resources to lead healthy and physically active lifestyles. Our findings suggest that competences for health-enhancing physical activity are unequally distributed in terms of both social and health-related factors. At the population level, therefore, strengthening movement competence, control competence, and self-regulation competence may be a relevant approach to promoting physical activity and addressing inequalities in physical activity participation. Future strategies should consider the different sub-competences as well as the specific contexts and living conditions of less physically active population groups. Beyond the national context, the present findings also contribute to current international efforts to strengthen competence-oriented approaches to physical activity promotion, including initiatives such as the Global Physical Literacy Action Framework [25].

## Supplementary Information

Supplementary Material 1.

## Data Availability

The data records can be obtained by submitting an application form through the Research Data Centre (Robert Koch Institute, MF4), which can be accessed at http://www.rki.de/fdz.

## Abbreviations

CASMIN: Comparative Analysis of Social Mobility in Industrial Nations
CI: Confidence interval
GEDA: German Health Update
PAHCO: Physical Activity-related Health Competence
PAHCO_12: Physical Activity-related Health Competence questionnaire
OR: Odds ratio
Ref: Reference group
RKI: Robert Koch Institute
WHO: World Health Organisation
