# Supplementary material for: Physical activity-related health competence differs by social and health-related factors: findings from a nationwide population-based study in Germany
Source: BMC Public Health. 2026 Aug 19;26:2397. doi: 10.1186/s12889-026-29096-0 (PMC13488593; doi:10.1186/s12889-026-29096-0)
Supplement: Supplementary file 1 [file 12889_2026_29096_MOESM1_ESM.docx]

Supplementary material

Table S1 PAHCO_12 items

| **Nr.** | **Attributable Subcompetence^1^** | **Item in English language (translated)** | **Item in German language (as used in PAHCO_12)** |
| --- | --- | --- | --- |
| 1 | Movement competence | I can maintain a slightly exhausting physical activity, which makes me breathe a little faster (e. g., walking, slow jogging or cycling), for 30 minutes without a break. ^a^ | Ich kann eine etwas anstrengende körperliche Aktivität, bei der ich etwas schneller atmen muss, z. B. Gehen, langsames Joggen oder Radfahren, 30 Minuten ohne Pause durchhalten.^a^ |
| 2 | Movement competence | I can lift an object that is approx. 10kg throughout several floors (e.g., full shopping bag, filled suitcase, small beverage crate).^a^ *[Interviewer note: In case of a request, please note: 3 floors]* | Ich kann einen ca. 10 kg schweren Gegenstand über mehrere Etagen hochtragen, z. B. volle Einkaufstasche, gefüllter Reisekoffer, kleine Getränkekiste.^a^ *[Interviewerhinweis: Bei Nachfragen erfolgt der Hinweis: 3 Etagen]* |
| 3 | Movement competence | I can stand on one leg and reach for an object without losing balance.^b^ | Ich kann auf einem Bein stehen, gleichzeitig nach einem Gegenstand greifen und verliere dabei nicht das Gleichgewicht.^b^ |
| 4 | Movement competence, control competence^1^ | My body awareness helps me to master physically demanding situations well.^b^ | Mein Körpergefühl hilft mir, körperlich fordernde Situationen gut zu bewältigen.^b^ |
| 5 | Control competence | I am able to adjust my training effort well to my physical condition.^b^ *[Interviewer note: The point is to choose the intensity of the training so that it suits your own physical condition. If people have never exercised before, please tick the answer category “does not apply”].* | Ich bin in der Lage, eine Trainingsbelastung gut auf meine körperlichen Voraussetzungen anzupassen.^b^ *[Interviewerhinweis: Es geht darum, die Intensität des Trainings so zu wählen, dass sie zu den eigenen körperlichen Voraussetzungen passt. Wenn Personen noch nie trainiert haben, dann bitte die Antwortkategorie „trifft nicht zu“ ankreuzen.]* |
| 6 | Control competence | I know how to use physical training to improve my endurance in the best possible way.^b^ *[Interviewer note: If people have never exercised, please tick the answer category “does not apply”].* | Ich weiß, wie ich mit körperlichem Training meine Ausdauerleistung am besten steigern kann.^b^ *[Interviewerhinweis: Wenn Personen noch nie trainiert haben, dann bitte die Antwortkategorie „trifft nicht zu“ ankreuzen.]* |
| 7 | Control competence | I know what to pay attention to in relation to my body in order to avoid excess load or insufficient load.^b^ | Ich weiß, worauf ich bei meinem Körper achten muss, damit ich mich nicht über- oder unterfordere.^b^ |
| 8 | Control competence | I am well able to work off pent-up stress and inner tension through exercise.^b^ | Ich kann aufgestauten Stress und innere Anspannung durch Bewegung gut abbauen.^b^ |
| 9 | Self-regulation competence, movement competence^1^ | I feel capable to perform physical activities that are challenging for me.^b^ *[Interviewer note: This is about the physical activity/movement that you find demanding or challenging].* | Ich traue mir zu, für mich anspruchsvolle körperliche Aktivitäten durchzuführen.^b^ *[Interviewerhinweis: Es geht um die körperliche Aktivität/Bewegung, die Sie für sich als anspruchsvoll empfinden oder die Sie vor eine Herausforderung stellt.]* |
| 10 | Self-regulation competence | When I decide to do more exercise, I am very disciplined in implementing this plan.^b^ | Wenn ich mir vornehme, mich mehr zu bewegen, habe ich viel Disziplin bei der Umsetzung.^b^ |
| 11 | Self-regulation competence | I am very able to choose from a variety of exercise and sport activities the one that suits me best.^b^ | Ich bin sehr gut in der Lage aus einer Vielfalt von Sport- und Bewegungsangeboten das auszuwählen, welches am besten zu mir passt.^b^ |
| 12 | Self-regulation competence | If I have planned to exercise, I generally follow through on this plan.^b^ | Wenn ich eine sportliche Aktivität geplant habe, setze ich das in der Regel auch um.^b^ |

^a^ Response options (items 1+2): 1 = “I can do this without any problems” [German: „Ich kann dies ohne Probleme”], 2 = “I have some slight problems” [„Ich habe leichte Probleme”], 3 = “I have moderate problems” [„Ich habe mäßige Probleme”], 4 = “I have major problems” [„Ich habe große Probleme”], 5 = “I cannot do this” [„Ich kann dies nicht”].^b^ Response options (items 3-12): 1 = “does not apply” [German: „trifft nicht zu”] … 5 = “applies very much” [„trifft sehr zu”].

Table S2 Correlation matrix (*ρ)* of considered influencing factors of PAHCO (GEDA 2023, n = 3,734)

| **Variable** | **1. Gender** | **2. Age group** | **3. Education** | **4. Income** | **5. Self-assessed health status** | **6. Chronic disease** | **7. Self-assessed mental health** | **8. Social support** |
| --- | --- | --- | --- | --- | --- | --- | --- | --- |
| **1. Gender** | 1.00 |  |  |  |  |  |  |  |
| **2. Age group** | 0.06* | 1.00 |  |  |  |  |  |  |
| **3. Education** | -0.04* | -0.07* | 1.00 |  |  |  |  |  |
| **4. Income** | -0.08* | -0.05* | 0.34* | 1.00 |  |  |  |  |
| **5. Self-assessed health status** | -0.04* | -0.22* | 0.18* | 0.19* | 1.00 |  |  |  |
| **6. Chronic disease** | -0.06* | -0.16* | 0.08* | 0.09* | 0.43* | 1.00 |  |  |
| **7. Self-assessed mental health** | -0.09 | -0.08* | 0.17* | 0.19* | 0.41* | 0.25* | 1.00 |  |
| **8. Social support** | 0.08* | -0.02 | 0.10* | 0.12* | 0.17* | 0.10* | 0.24* | 1.00 |

*p<0.05, n – complete cases considered in the regression models

Table S3 Global tests of interaction effects (GEDA 2023, n =3,734)

| Outcome | Interaction tested^a^ | Design-based F (df1, df2) | p |
| --- | --- | --- | --- |
| PAHCO overall | Gender × age group | 1.08 (3, 3731) | 0.356 |
| Movement competence | Gender × age group | 1.32 (3, 3731) | 0.267 |
| Control competence | Gender × age group | 0.94 (3, 3731) | 0.421 |
| Self-regulation competence | Gender × age group | 1.81 (3, 3731) | 0.142 |
| PAHCO overall | Age group × education | 0.87 (6, 3728) | 0.518 |
| Movement competence | Age group × education | 1.58 (6, 3728) | 0.149 |
| Control competence | Age group × education | 1.38 (6, 3728) | 0.219 |
| Self-regulation competence | Age group × education | 0.91 (6, 3728) | 0.484 |

^a^ Each interaction was examined separately in a weighted logistic regression model adjusted for gender, age group, educational attainment, Equivalent household income, self-rated health, chronic disease, mental health and social support. No interaction effect reached statistical significance at p < 0.05, p-values are based on joint design-based Wald tests of all interaction terms, df (numerator, denominator) – degrees of freedom.

**Table S4** Multiple regression with the same predictors but metric PAHCO outcomes as part of a robustness analysis (GEDA 2023, N = 3,734).

|  |  |  | PAHCO (metric scores) | | |
| --- | --- | --- | --- | --- | --- |
| **Variable** |  | PAHCO overall score β | Movement competence β | Control competence β | Self-regulation competence β |
| **Gender** | Female | **−0.048*** | −0.093*** | −0.019 | −0.014 |
|  | Male | *ref.* | *ref.* | *ref.* | *ref.* |
| **Age group** | 18–29 years | *ref.* | *ref.* | *ref.* | *ref.* |
|  | 30–44 years | −0.010 | −0.004 | 0.004 | −0.036 |
|  | 45–64 years | −0.087** | −0.144*** | −0.028 | −0.062 |
|  | 65+ years | −0.179*** | −0.295*** | −0.104** | **−0.069*** |
| **Education** | Low | −0.165*** | −0.169*** | −0.168*** | **−0.097**** |
|  | Medium | **−0.041*** | −0.055** | −0.050** | 0.001 |
|  | High | *ref.* | *ref.* | *ref.* | *ref.* |
| **Equivalent income in quintiles** | 1^st^ Quintile (lowest) | −0.083* | −0.073* | −0.100** | −0.044 |
|  | 2^nd^ Quintile | 0.008 | 0.011 | −0.006 | 0.022 |
|  | 3^rd^ Quintile | −0.066** | −0.033 | −0.068** | −0.074** |
|  | 4^th^ Quintile | −0.003 | −0.008 | −0.014 | 0.020 |
|  | 5^th^ quintile (highest) | *ref.* | *ref.* | *ref.* | *ref.* |
| **Self-rated general health** | Very bad/bad/fair | −0.283*** | −0.305*** | −0.226*** | −0.218*** |
|  | Good/very good | *ref.* | *ref.* | *ref.* | *ref.* |
| **Chronic condition** | Yes | −0.081** | −0.114*** | **−0.033** | −0.074** |
|  | No | *ref.* | *ref.* | *ref.* | *ref.* |
| **Self-rated mental health** | Poor/fair | −0.198*** | −0.170*** | −0.213*** | −0.138*** |
|  | Good | −0.117*** | −0.074*** | −0.136*** | −0.098*** |
|  | Very good/excellent | *ref.* | *ref.* | *ref.* | *ref.* |
| **Social support** | Poor | −0.124*** | −0.075** | −0.133*** | −0.122*** |
|  | Moderate | −0.019 | 0.008 | −0.030 | −0.028 |
|  | Strong | *ref.* | *ref.* | *ref.* | *ref.* |
| **Model fit** | R² | 0.395 | 0.469 | 0.302 | 0.210 |

**Notes:** β denotes the standardized regression coefficient from weighted linear regression models with robust standard errors. Negative β values indicate lower metric competence scores compared with the reference category (ref.). R² (coefficient of determination), indicating the proportion of variance explained by the model. Differences in significance level to the main models (Table 4) are marked in bold. * p < 0.05; **p < 0.01; ***p < 0.001
.
